# Reply: Glucocorticoid use and skin cancers

**DOI:** 10.1038/sj.bjc.6601222

**Published:** 2003-08-26

**Authors:** M R Karagas, D W Nierenberg

**Affiliations:** 1 Departments of Community and Family Medicine, Medicine, Pharmacology and Toxicology, and the Norris Cotton Cancer Center, Dartmouth Medical School, Lebanon, NH 03756, USA

**Sir**,

We appreciate Dr Purdue's interest in our report on use of glucocorticoids and skin cancers. As suggested in his letter, we reclassified indication for steroid use into two categories: (1) atopic conditions (i.e. ‘respiratory conditions and asthma’ and ‘allergies’) and (2) non-atopic conditions (i.e. ‘musculoskeletal and connective tissue disease’, ‘neoplasm’, ‘gastrointestinal disease’, and ‘other condition’) (Table 1

**Table 1:** Oral glucocorticoid use according to indication among skin cancer cases and controls

**Steroid use^a^**	**Indication**	**Controls *N* (%)**	**BCC *N* (%)**	**SCC *N* (%)**
No		491 (95.0)	534 (93.0)	245 (91.1)
Yes	Any use^b^	26 (5.0)	40 (7.0)	24 (8.9)
	Treat possible atopic conditions^c^	11 (2.1)	12 (2.1)	9 (3.4)
	Treat possible non-atopic conditions^d^	15 (2.9)	28 (4.9)	15 (5.6)

BCC=basal cell carcinoma; SCC=squamous cell carcinoma.

a Use of oral glucocorticoid use for 1 month or longer.

b Excludes three individuals with more than one indication (two BCC, one SCC) and two individuals with missing data on indication for use (one BCC, one SCC).

c Includes ‘respiratory conditions and asthma’ and ‘allergies’.

d Includes ‘musculoskeletal and connective tissue disease’, ‘neoplasm’, ‘gastrointestinal disease’, ‘other condition’.

). In the reanalysis of the data, we noted an error in Table 1 of the original report. There were only two controls that used glucocorticoids for allergies (the seven controls in the original report included users of inhaled steroids). The correct numbers appear in Table 1 below and are restricted to oral users. The odds ratios associated with oral glucocorticoid use, stratified by Dr Purdue's classification, are shown in Tables 2

**Table 2:** Association between oral glucocorticoid use and BCC, with further analysis by indication

		**BCC**	**BCC**
**Steroid**		**Crude**	**Age, sex adjusted**
**use^a^**	**Indication**	**OR (95% CI)**	**OR (95% CI)**
No		1.00 (reference)	1.00 (reference)
Yes	Any use^b^	1.45 (0.87, 2.41)	1.43 (0.86, 2.38)
	Treat possible atopic conditions^c^	1.00 (0.44, 2.29)	0.97 (0.42, 2.23)
	Treat possible non-atopic conditions^d^	1.72 (0.91, 3.25)	1.70 (0.90, 3.24)

BCC=basal cell carcinoma; OR=odds ratio; CI=confidence interval.

a Use of oral glucocorticoid use for 1 month or longer.

b Excludes two cases with more than one indication and one case with missing data on indication for use.

c Includes ‘respiratory conditions and asthma’ and ‘allergies’.

d Includes ‘musculoskeletal and connective tissue disease’, ‘neoplasm’, ‘gastrointestinal disease’, ‘other condition’.

and 3

**Table 3:** Association between oral glucocorticoid use and SCC, with further analysis by indication

		**SCC**	**SCC**	**SCC**
		**Crude**	**Age, sex adjusted**	**Age, sex, skin type^b^adjusted**
**Steroid use^a^**	**Indication**	**OR (95% CI)**	**OR (95% CI)**	**OR (95% CI)**
No		—	—	—
Yes	Any use^c^	1.93 (1.09, 3.41)	1.98 (1.10, 3.57)	2.21 (1.21, 4.04)
	Treat possible atopic conditions^d^	1.64 (0.67, 4.01)	1.68 (0.67, 4.19)	1.75 (0.68, 4.46)
	Treat possible non-atopic conditions^e^	2.00 (0.96, 4.17)	2.10 (0.99, 4.47)	2.49 (1.16, 5.36)

SCC=squamous cell carcinoma; OR=odds ratio; CI=confidence interval.

a Use of oral glucocorticoid use for 1 month or longer.

b Skin reaction to sun exposure.

c Excludes one case with more than one indication and one case with missing data on indication for use.

d Includes ‘respiratory conditions and asthma’ and ‘allergies’.

e Includes ‘musculoskeletal and connective tissue disease’, ‘neoplasm’, ‘gastrointestinal disease’, ‘other condition’.

. The results for ‘any use’ differ very slightly from the original report because we removed individuals with more than one indication or with missing data on the indication for use for this reanalysis.

The data for basal cell carcinomas (BCC) provide some support for his hypothesis, that is, the association may be limited to non-atopic conditions (Table 2). However, lack of precision in our risk estimates prevents us from drawing any firm conclusions. In our original report, the association was stronger for squamous cell carcinomas (SCC), and for this cell type, the risk associated with oral glucocorticoid use does not differ much by type of condition (Table 3). Unfortunately, we were unable to perform the recommended analysis of inhaled steroid use since the indications for use almost exclusively fell into the atopic category (Table 4

**Table 4:** Indication for glucocorticoid use among those who use inhaled steroids

**Indication for steroid use**	**Inhaled only**	**Inhaled+oral^a^**
Respiratory conditions and asthma	29	10
Allergy	7	1
Musculoskeletal and connective tissue disease	1	0
Gastrointestinal disease	0	1
Other condition	3	2

a Three people have more than one reason for steroid use.

). The potential effect of immune response and immunosuppression on the incidence of skin cancer is of considerable interest to us. We hope that further studies will address commonly used immune-modulating drugs along with atopic conditions, and that this, in turn, will shed light on this pathway to human malignancies.

